# Stopping Antidepressants and Anxiolytics as Major Concerns Reported in Online Health Communities: A Text Mining Approach

**DOI:** 10.2196/mental.7797

**Published:** 2017-10-23

**Authors:** Adeline Abbe, Bruno Falissard

**Affiliations:** ^1^ Centre de recherche en épidémiologie et santé des populations (CESP)/ Institut national de la santé et de la recherche médicale (INSERM) U1018 Maison de Solenn Paris cedex 14 France

**Keywords:** social media, antidepressant, anxiolytic, text mining, data mining

## Abstract

**Background:**

Internet is a particularly dynamic way to quickly capture the perceptions of a population in real time. Complementary to traditional face-to-face communication, online social networks help patients to improve self-esteem and self-help.

**Objective:**

The aim of this study was to use text mining on material from an online forum exploring patients’ concerns about treatment (antidepressants and anxiolytics).

**Methods:**

Concerns about treatment were collected from discussion titles in patients’ online community related to antidepressants and anxiolytics. To examine the content of these titles automatically, we used text mining methods, such as word frequency in a document-term matrix and co-occurrence of words using a network analysis. It was thus possible to identify topics discussed on the forum.

**Results:**

The forum included 2415 discussions on antidepressants and anxiolytics over a period of 3 years. After a preprocessing step, the text mining algorithm identified the 99 most frequently occurring words in titles, among which were escitalopram, withdrawal, antidepressant, venlafaxine, paroxetine, and effect. Patients’ concerns were related to antidepressant withdrawal, the need to share experience about symptoms, effects, and questions on weight gain with some drugs.

**Conclusions:**

Patients’ expression on the Internet is a potential additional resource in addressing patients’ concerns about treatment. Patient profiles are close to that of patients treated in psychiatry.

## Introduction

The different Internet resources make it possible to share content quickly and to interact within a large population. The biomedical literature shows a significant increase in studies published in online communities. In 2015, almost 400 publications linking to Facebook, over 300 linking to Twitter, and almost 400 documents linking to blogs and forums were published. Online health communities (OHCs), social media, and blogs are a potential mine of information exchanged daily. Among the applications of text mining, the automatic analysis of data from the Internet is a challenge. In fact, the large amount of data available on this platform can be processed by the tools of natural language processing. In addition, screening the Internet is almost impossible manually, and it is a very interesting application for automatic data extraction tools [1].

Social networks are a particularly dynamic way to capture the concerns of a population in real time. Blogs, microblogs such as Twitter [2], social networking sites such as Facebook [3], and discussion forums are spaces for exchange of information where people publish their personal stories or their opinions in real time. This dynamic and continuously updated source of information is ideal for the collection of data in a variety of disciplines, enabling users to tap into the wisdom of crowds. Through the Internet, we could explore the information exchanged, irrespective of its quality. It is important to be aware of information disseminated on the Internet. It is useful to know what concerns people have, as well as to inform, alert, correct, and prevent specific issues.

The online social networks provide a valuable complement to communication face-to-face and help patients to improve their self-esteem and social skills [4-6]. Social networks encourage patients to be more active in their social environment [7]. For example, patients can chat via online media about their private problems without fear of prejudice or discrimination [8]. The impact on patient health of sharing information on the Internet is a topic that has been explored in the literature. Yan and Tan investigated the usefulness of OHC on patient health [9]. The authors found that patients benefit from the experience of others and that their participation in the online community helped to improve their health. Social support exists in various forms and depends on patients’ health conditions. However, one factor remains essential whatever the illness: emotional support plays a very important role in helping patients improve their health.

### Social Media as a Resource for Mental Health Service

Some studies have focused on the use of the social media and Internet psychiatry forums. OHCs and the social media as a resource for mental health service users are important in reducing stigma and promoting help-seeking behaviors. The analysis of the information shared on the Web is crucial in psychiatry, as public misinformation could negatively affect mood. Research on the social networks has increased and has explored different aspects of people’s health status. Several studies have considered the way depression and eating disorders are discussed on Twitter [10], whereas others have focused on the detection of depression via identification of events, emotions, and negative thoughts in Web and Facebook messages [11-13], or on suicide detection on Twitter [14], or again on exploring disorders such as depression by analyzing the behaviors of Facebook users [15]. With the growing popularity of the social media, the impact of support on the Internet is of interest only because it naturally occurs outside the setting of professional guidance.

The influence of the social networks has been studied for the development of attitudes of mutual trust and self-help. In some cases, face-to-face support cannot provide adequate help for patients with mental disorders [16]. The proliferation of OHCs has enabled patients to share information and experiences and to communicate on their illness. Ma X found that social interaction in online communities such as PatientsLikeMe.com was significantly associated with time to recovery in patients with mental disorders [17]. However, online social interactions reveal a more complex picture. Several studies have linked the use of OHCs to decline in mood, well-being, and quality of life [18-21]. For example, the passive consumption of OHCs’ content with no active involvement has been linked to a reduction in social interactions in real life and increased solitude [22]. This finding reflects a limitation of online interactions with respect to social activity in real life. The impact of Internet on behavior cannot be ignored. An obvious example is the impact of the use of Facebook combined with comparisons of physical appearance online, which could lead to more disordered eating habits and associated conditions [23].

### Applications of Text Mining to Web Data

Several studies have been published on the applications of text mining to Web data. The exploitation of Internet data has provided early monitoring information on adverse reactions to drugs [24-26]. The study of social interactions on the Web in real or near-real time is also of interest in public health surveillance. In a health crisis, as experienced with the spread of the Ebola or influenza A (H1N1) viruses, it is important to understand the expectations and questions of the population [27-29]. These analyses provide content to inform health authorities to anticipate epidemics such as H1N1 and to respond to the concerns of the public. Other studies have used this source of information to investigate depressive trends from messages posted on the Web [13]. The purpose of the exploration of data from blogs is, in this case, to help bloggers or authors of posted messages by detecting major depressive disorders early. More generally, the objective is to capture patient perceptions through the messages posted on discussion forums. The patient perspective includes views on treatment, on the illness, and on priorities and needs in terms of health [30-32].

Text mining is proving to be a powerful method to exploit large continuous flows of user-generated content on the World Wide Web. This resource has rarely been exploited to understand the perceptions of patients about treatments. We set out to study an online discussion forum dedicated to the use of antidepressants and anxiolytics to explore patient concerns.

## Methods

### Data Collection

Our dataset is derived from a French online discussion forum about drugs, illness, procedures, and other information relating to general health. As mentioned in the forum charter, discussions can be read and potentially used by all. We focused initially on the titles of discussions from 2013 to 2015. The participants themselves summarize the topic or question they post on the forum. In other words, we focus on a condensed form of the concerns of the participants regarding antidepressants or anxiolytics.

The data extraction step is dependent on the data source and differs according to whether it is data from a website, from patient medical records, or from qualitative interviews. In our study, health information was extracted from Web pages via a program that explores the Web of data using R packages (R Project for Statistical Computing). Our corpus of documents was formed from discussion titles. A page contains 50 topics and each topic consists of a title, an initial message (demand), and potential responses (messages). To create this database, we implemented the following process: first, we extracted pages including lists of discussions. Links to each discussion are found on the website in a specific location. The storage address for a discussion is indicated via a URL link. By capturing all the discussion addresses, we had access to the messages stored there in Hyper Text Markup Language (HTML) format. Second, each URL and each of the discussions were analyzed to remove unnecessary information (images and advertising) and to extract the date, titles, and discussion of messages in a Microsoft Excel file.

There has been discussion about the ethical concerns of analyzing data retrieved from OHCs [33]. To minimize these concerns, we searched for and included only the title of discussions that were publicly available. We used the dataset solely for statistical analysis and reporting of aggregated information and not for investigation of specific individuals or organizations. We have no prior knowledge of the possible identities of any study participants. We did not submit our study to an ethics committee because no user was interviewed. The identity of users is protected because of the use of alias, and all usernames or potential demographic characteristics in the results were removed from the dataset.

### Preprocessing Step

Once the extraction of data is performed, the data preparation stage can begin. The tools need to be adapted to the language (English, French, and Spanish) and to the vocabulary if certain words are used in a specific domain (eg, medical). In addition, words used on the Internet via social networks or blogs are not the same as those used in a newspaper. We, therefore, need to pay special attention to spelling irregularities and to include everyday words used in spoken language.

A morphological analysis is the first part of the process. It consists of analyzing the morphology of the sentences in the text. All messages are reviewed by screening for particular typographic elements such as accents. This step is essential in French because accents are a characteristic of our language. For example, the letter “a” can have several variants and is replaced by its generic form without an accent. Then, there is a harmonization step, consisting of converting all lowercase letters to uppercase. Each sentence is finally cut off using the punctuation that defines it. Punctuation marks are deleted. Finally, the spaces between the words are used to delineate them.

The next step is to parse the text and to remove noninformative elements such as numbers or link words occurring in the database. The words and codes for data extraction from the Internet may also be present, such as XML and HTML tags (< html>, </ n>...). Finally, a list of “stop words” is predefined in the software to automatically delete the list of prepositions and articles ( what, my, ...) that are not informative and to reduce the list of words that are most relevant to the analysis.

The last step is stemming, which consists of grouping similar words according to a common root. For instance, a verb can have different spellings following conjugation rules. Stemming enables us to group every inflection of the verb into one term, which is the root. For instance, the word “continue” exists in different variants—“continued,” “continuing,” “continuous,” “continuation,” and so on. The three inflected forms will be identified as one relating to “continue.” The same principle is applied for compound words or words with a prefix or suffix. To perform the stemming step, it is necessary to have an exhaustive list of words including all variants and the associated root. The quality of this processing varies with the software used and from one language to another and depends on the list of words referenced. Finally, to simplify the analysis of the treatments mentioned, we harmonized the names of the different drugs by using their international nonproprietary names.

Initially, every word in every sentence is recognized as unique. At the end of this stage of data preparation, the number of words is reduced following the simplification of word variants. To analyze the occurrence of these words in our corpus, a contingency table is created and called document-term matrix (DTM). In our study, we analyzed only the words used in the titles of discussions. Our DTM table shows the number of times a word was used (column) in a discussion title (online). The majority of words appear only in some titles. Accordingly, if a DTM is still almost empty, it means that there are a large number of 0s in the table. We, therefore, need to adapt the data modeling approach to this type of data.

### Analysis

#### The Most Frequent Words

The easiest and commonest way to visualize textual data is the word cloud. The aim is to display each word and represent its frequency by the size of the font used. First, only words included in the DTM table are used. Then, the frequency of each word is calculated, and the list is ordered in decreasing manner. The word that appears most frequently is represented with the largest font. The second word is most often graphically smaller than the first word but larger than the third word in the list and so on with other words. In the end, the word cloud reflects the word frequency table, maximizing the visibility of the most common terms.

#### Centrality of Co-occurrences

To analyze the patterns of occurrence of words in the discussion titles, we studied the influence of each word in terms of co-occurrence. Due to the sparsity of the DTM, correlation analysis was not appropriate. The analysis of co-occurrences, via centrality measures using graph theory, is an alternative, which has been proved to be better in quantity and quality [34]. The patterns of word occurrences can be graphically represented in two complementary forms inspired by the graph theory and social network analysis.

The first type identifies a centrality pattern, which highlights some words of importance based on their better positioning in the co-occurrence relationships. These words have a central role in some units in the graph. Centrality can be measured by a local measure using the degree of centralization, considering that words with many connections are the most important words. The degree of centrality measures the importance of a word and is involved in a large number of interactions, measuring by an exposure index to what is flowing through the network. Another way of looking at centrality is by considering how important words are in connecting to other words (betweenness centrality). The idea is to reflect the mediation role of words based on how many words each word would have to go through to reach the others.

#### Community of Co-occurrences

The second type identifies a modular pattern of occurrence (community), where the words are grouped into classes based on semantic similarities (ie, similar semantic patterns of word occurrences). The aim of this analysis was to identify the thematic structure of the text [35,36]. This analysis yields a division into classes and a hierarchy of words based on co-occurrences. A graph shows words, each being linked by ties of co-occurrence. By construction, words in the same class are interconnected and connected to another class based on co-occurrence links in the titles. We present only results from the fast greedy algorithm based on the high density of internal links of words inside a group [37]. One study indicates more stable and better results with fast greedy algorithms compared with others such as k-means, expectation maximization, and the walktrap algorithm [38]. All analyses were performed using R tm package.

## Results

### Data Collection

The Health Forum studied is a French-language website, Doctissimo [39], and includes 2415 discussions on antidepressants and anxiolytics from 2013 to 2015. It includes 33,865 messages written by 1257 different authors. On average, a first message posted (a question) received 14 responses. In 7.7% of cases (n=185), questions received no answer on the forum. In other cases, a demand can be widely discussed with up to 50 replies. The average time of discussion is 30 days. A discussion can be maintained over a longer period with interruptions of up to several years.

### Preprocessing Step

**Figure 1:**
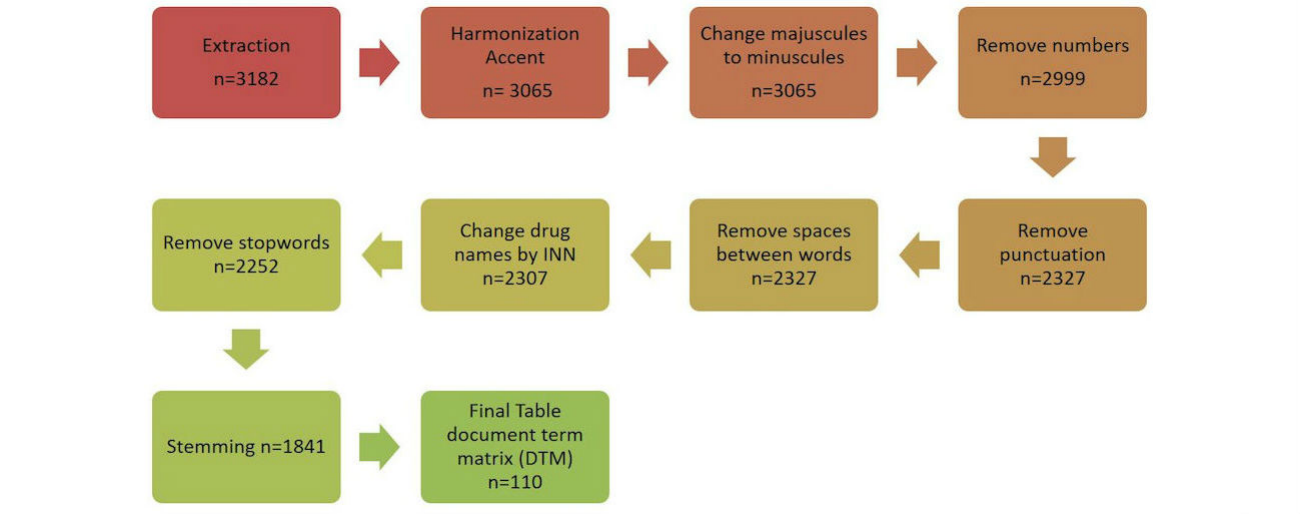
Preprocessing step.

The preprocessing step is represented in Figure 1, showing how text data are structured. Each step of preprocessing is shown, as well as the impact of each step on reducing the number of words stored in the DTM final table.

The titles of discussions extracted initially contained 3025 different words. After the pretreatment step, only 99 words were identified as being the most representative, in other words, only the words that appeared most frequently in the titles and considered the most informative (excluding prepositions, articles, and some adverbs).

Finally, the final table reduction step was applied to remove words that appeared infrequently. We did not analyze all the terms in the titles because many words are not informative. To reduce the size of our final DTM table without the risk of losing information, we removed the words occurring in less than 0.05% of the titles. Few words are retained as the most relevant by text mining to define the title content. Content of some of the titles was reduced to one or two words. More than 400 titles did not contain any of the words listed by text mining in the DTM as the most frequent. Several reasons could explain this phenomenon. First, some titles could include some uncommon words. Second, noninformative words are deleted during the preprocessing phase.

### Analysis

#### The Most Frequent Words

**Figure 2:**
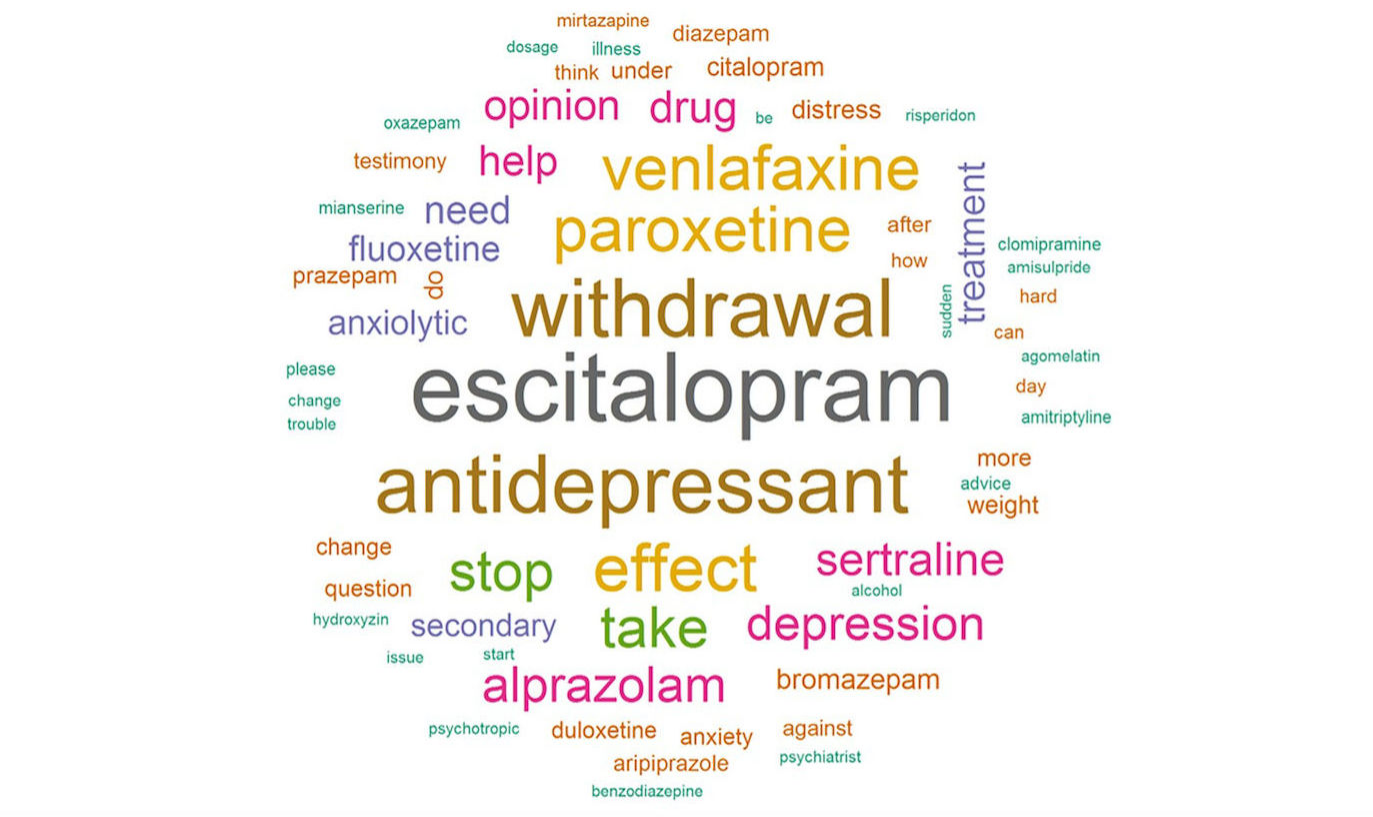
Wordcloud.

Figure 2 is the word cloud that visually represents word frequencies in the data. Letter size is proportional to the frequency of the words in the discussion titles. The more often the word appears in the titles of discussions, the larger the font.

Words related to antidepressants were the most frequent. The words corresponding to information sharing between participants, such as “help,” “testimony,” “advice,” “need,” and “opinion” are also present. Multimedia Appendix 1 lists the 20 most frequent words by decreasing order plotted in the word cloud. The drug names “escitalopram” and “venlafaxine” are words that are frequently used in the titles of discussions and to a lesser extent the drugs fluoxetine, sertraline, alprazolam, paroxetine, and bromazepam. The list of 26 molecules named in the discussions is presented in Multimedia Appendix 2. Other related words such as *stop* and *take treatment* were often used. In Figure 1, we can see that symptoms relative to weight, anxiety, depression, and distress are mentioned. These symptoms are more difficult to identify automatically because several denominations can be used to describe the same condition.

**Figure 3:**
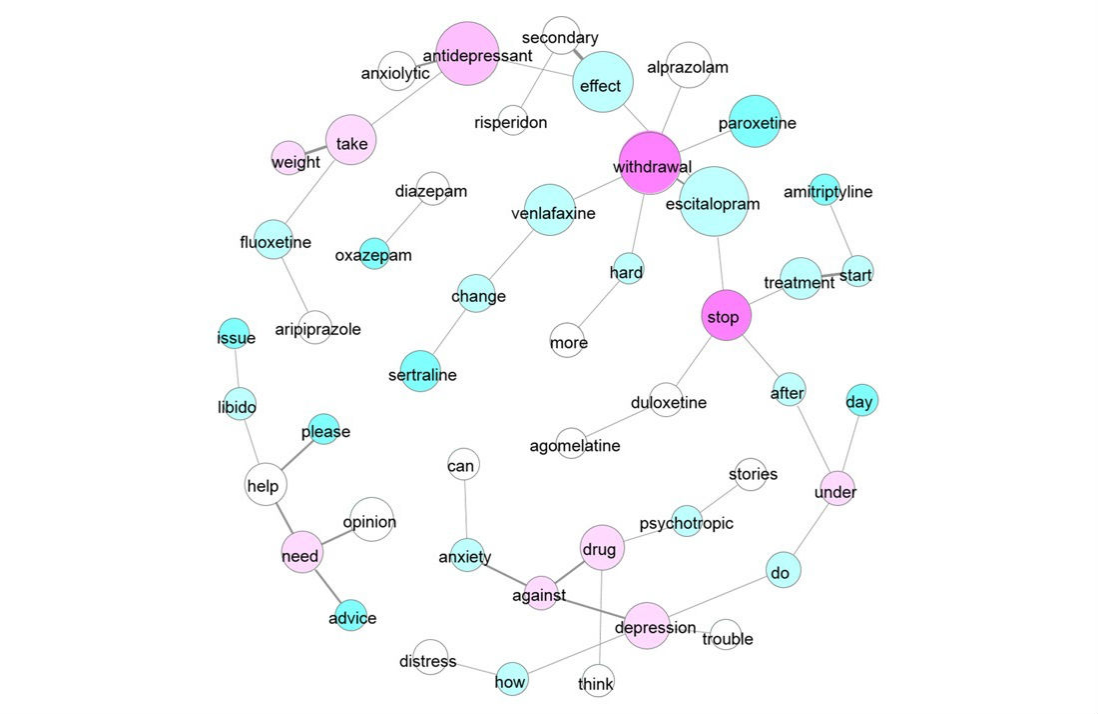
Centrality of co-occurrences based on degree algorithm.

**Figure 4:**
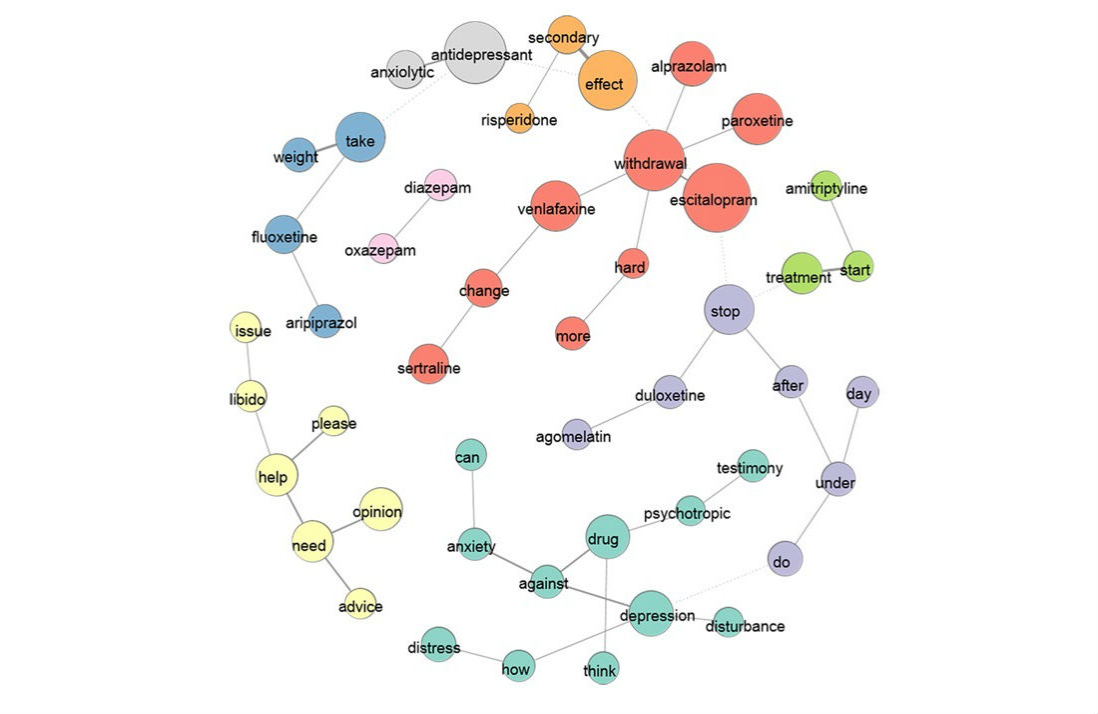
Cluster of co-occurrences-community detection based on modularity (fast greedy algorithm).

#### Centrality of Co-occurrences

Centrality reflects the relative importance of a word within a corpus (ie, the links between words by measuring the position of a word in the network). The centrality measure based on degree enables visualization of the most frequently used words in the forum. Figure 3 shows the words considered the most central, in the sense that they have numerous links to other words (in pink).

As in the word cloud, the most popular words are “withdrawal,” “stop,” and “antidepressant.” These words reflect major concerns expressed in the forum. Betweenness centrality relates to words with a mediator role, serving as paths linking to other words in the network. It quantifies the control of a word on the communication between other words. Six words are considered as mediator-linking terms relative to the request (“after,” “under,” and “do”) and defining different topics around common terms (“escitalopram,” “withdrawals,” and “antidepressant”).

#### Community of Co-occurrences

The detection of “communities” makes it possible to highlight patterns of co-occurrences, nonhierarchical but localized. Community detection based on modularity (fast greedy algorithm) is used to visualize different topics in Figure 4.

Nine clusters of words are identified, representing the interconnection of terms on the basis of their co-occurrence in titles:

- Depression, distress, and anxiety, where people ask about the experiences of people who took treatments against these symptoms (11 words in turquoise)
- Withdrawal linked to paroxetine, escitalopram, alprazolam, and changes of treatment especially with venlafaxine and sertraline (9 words in red)
- Effects after stopping medication of duloxetine and agomelatine (7 words in violet)
- Search for advice, assistance, and libido issues (7 words in yellow)
- Weight gain with fluoxetine and aripiprazole (4 blue words)
- Effects of amitriptyline (3 words in green)
- Side effects of risperidone (3 words in orange)
- Changing prescription and switching two antidepressants: duloxetine and agomelatine (2 green words)
- Concerns about the effects and side effects of medications (2 gray words)

Detecting communities is an interesting graphic approach to visualize knowledge of relational data and to bring information to light more quickly when it is hidden in large volumes of data. Similar results were found using the random walk algorithm (walktrap).

## Discussion

### Principal Findings

The principal concerns in the forum relate to withdrawal and discontinuing certain antidepressants. We can see the central role of withdrawal in patients’ questions. This issue was previously minimized for a long period. In 1997, a survey concluded that many physicians denied being aware of the existence of antidepressant withdrawal symptoms [40]. The incidence of discontinuation reactions is unclear, owing to the lack of research and a clear definition of withdrawal [41]. Conclusions from conventional approaches such as meta-analyses and those from our text mining on an online forum are consistent. Events previously reported with antidepressants after discontinuation of treatment for major depression are nausea, vomiting, diarrhea, headache, dizziness, insomnia, sexual side effects, and weight gain [42]. For instance, 31% of nausea was reported by patients with major depression. Adverse event profiles varied with the drugs. However, only 13% of clinical studies collected adverse events using a standardized scale. The lack of guidance based on evidence available to both practitioners and patients reflects a lack of information on how to deal with discontinuation of antidepressant medication [36].

### Interest in Analyzing Online Health Communities

The study of interactions on OHCs provides an additional source of information to better understand the difficulties encountered in real life. Patients may be able to develop skills to overcome the difficulties of communication and recovery. In our study, we identified patients’ need to share experiences about illness management and to brighten their lives through social interactions on these online platforms [9]. The online social media thus plays a complementary role to that of the traditional mental health services and helps patients understand their conditions more fully and take better control of their illness and behavior [43]. For example, although many treatment decisions are still based on empirical judgments that might not have solid evidence to support them, sharing health care information on OHCs can enable patients to perceive their illness from another point of view, do their own research online, and make their own informed decisions about how to manage their illness [44-46]. Patients consult various online sources, in particular when they feel that their physician does not meet their information needs during a consultation. Concerns about topics discussed on the forum, such as withdrawal, weight gain, or dosage, need to be asked directly to a professional health care provider. Encouraging communication with a physician would help to clarify what “withdrawals” is referring to. The word “withdrawals” could be used inadequately by a forum and used to define two concepts: (1) the classic antidepressant discontinuation syndrome and (2) withdrawal syndrome relative to benzodiazepines use. In our study, we considered both antidepressants and anxiolytics, and the difference between the two technical words is probably not well established in the forum. However, two terms (withdrawals and stop) relative to the same concern are frequently reported in the title of discussion, reflecting a major preoccupation in the forum. The perceived quality of communication with physicians is one of the factors influencing the use of the Internet as a source of information [47].

### Limitations

We focus our analysis on people posting messages via the Internet, meaning that they have Internet access. There are still many people who do not use the Web on a regular basis. In these communities, there may be no easy way to obtain general health information, and we cannot therefore extrapolate our results to the views and behaviors of other population. However, Internet usage is increasing exponentially with technology and connectivity ever more widely available. There is, therefore, a need to monitor the changing demographics of website users (geographical location, age, and gender). In addition, not all information has the same impact on the Internet, and certain factors can quantify their influence on patient behavior [48]. Information quality, emotional support, and credibility of the source have a significant, positive impact on the adoption of health information. Among these criteria, the quality of information plays an important role in shaping patient decisions.

### Ethics

For the moment, no guideline is available to inform how to deal with data ownership. Although there are potential benefits of OHCs’ content analysis, it introduces new ethical challenges. The lack of clear guideline to conduct online human subject’s research leaves researchers with no clear way to analyze data shared on the Internet [49]. Only two reports provide advice on psychological research online in the American Psychological Association website [50]. In 2002, one report produced by the Board of Scientific Affairs Advisory Group on conducting research on the Internet identified the opportunities and challenges of conducting research on the Internet. However, their suggestions could not be adapted for new way communication tools such as OHCs. In 2012, a second report written presented ethical dilemma of subject research on the Internet. No recommendation of any guidelines beyond the requirement that any research conducted on the Internet has been proposed.

Consequently, this gap discourages social scientists from conducting online research. Several options have been used by researchers to publish their research based on Internet data. Some scientists do not publish any information about ethics consideration. Computer scientists raise fewer concerns because they are often unfamiliar with ethical and social implications. One study using Twitter data asked the advice of an institutional review board. They qualified the project as not human subjects’ research because public identification handles are avatars and are not identifiable living individuals according to local and national regulations [51]. In another study, the authors considered it as a post hoc analysis and explained that no ethical approval or informed consent is needed [52]. Website terms and conditions indicate that we should contact the website to obtain an agreement to use data hosting in their platform. In our study, we contacted the forum’s owner to present our project and to obtain their agreement to use their data. Different approaches of ethical considerations using Internet content are needed and would implicate discussion to define a clear guideline between OHCs, institutional review boards, and researchers. Few previous studies publishing results based on Internet user analysis report a section ethics statement. In this case, authors mentioned that data collection process has been carried out through the Facebook or Twitter API, which is publicly available, and only public available data were used for the analysis. We recommend reading attentively the conditions of utilization that might be different on each website and contact them to explain the research project avoiding any potential issues.

Despite these limitations, the antidepressants and anxiolytics cited are coherent for the management of patients with depression. Escitalopram, paroxetine, venlafaxine, and sertraline are the main antidepressants used in practice to treat depressed patients in France [53]. The French population is well known to be a major consumer of anxiolytics, but the majority of drugs reported in the forum are antidepressants. Antidepressants are more often mentioned than benzodiazepines in the titles of discussions. Prescriptions of anxiolytics such as Lexomil and Xanax decreased by 1.42% in 2014. The trend of benzodiazepines continued to decrease in 2015 compared with previous years. However, prescriptions of antidepressants increased by 0.67% in the same period and could explain why benzodiazepines are less frequent in discussion titles.

### Conclusions

Our analysis focuses on the most frequent words used in 2415 titles on a French online forum about antidepressants and anxiolytics. Major concerns addressed in the titles are as follows: (1) stopping medications (using the word “withdrawal”) for certain antidepressants, (2) the need to share the experience of symptoms (depression and anxiety), (3) effects, and (4) questions concerning weight gain with some treatments. The analysis of centrality gives a general idea of the words used. In addition, community analysis provides the context of the use of these words, helping to identify questions discussed in the forum. Our findings show that the patient profiles asking questions in the forum is close to that of patients treated in psychiatry. The concerns expressed are coherent with real-life situations and are not outlandish requests and complaints about mental health issues. Our research is based on text mining tools that are more and more used to evaluate drug surveillance. In practice, pharmacovigilance refers almost exclusively to spontaneous reporting systems. As a complement to the standard approach, analyzing what is spontaneously reported by patients could improve investigations in pharmacovigilance.

## Appendix

Multimedia Appendix 1 Top 20 most frequent words in titles.

Multimedia Appendix 2 Frequency of drug names in titles.

## Abbreviations

API: application program interface
DTM: document-term matrix
HTML: Hyper Text Markup Language
OHCs: online health communities
