# Supplementary material for: Stopping Antidepressants and Anxiolytics as Major Concerns Reported in Online Health Communities: A Text Mining Approach
Source: JMIR Ment Health. 2017 Oct 23;4(4):e48. doi: 10.2196/mental.7797 (PMC5673886; doi:10.2196/mental.7797)
Supplement: Multimedia Appendix 1 [file mental_v4i4e48_app1.pdf]

S1 Table. Top 20 most frequent words in titles

Rank	Words	Frequency	%
**1**	escitalopram	202	8.36
**2**	antidepressant	168	6.96
**3**	withdrawal	168	6.96
**4**	effect	137	5.67
**5**	paroxetine	129	5.34
**6**	venlafaxine	128	5.30
**7**	take	113	4.68
**8**	stop	110	4.55
**9**	alprazolam	97	4.02
**10**	depression	94	3.89
**11**	sertraline	88	3.64
**12**	drug	84	3.48
**13**	opinion	81	3.35
**14**	help	80	3.31
**15**	need	73	3.02
**16**	treatment	71	2.94
**17**	fluoxetine	65	2.69
**18**	anxiolytic	62	2.57
**19**	secondary	57	2.36
**20**	bromazepam	48	1.99
