# Supplementary material for: Stopping Antidepressants and Anxiolytics as Major Concerns Reported in Online Health Communities: A Text Mining Approach
Source: JMIR Ment Health. 2017 Oct 23;4(4):e48. doi: 10.2196/mental.7797 (PMC5673886; doi:10.2196/mental.7797)
Supplement: Multimedia Appendix 2 [file mental_v4i4e48_app2.pdf]

S2 Table. Frequency of drug names in titles

Rank	Words	Frequency	%
**1**	escitalopram	202	8.36
**5**	paroxetine	129	5.34
**6**	venlafaxine	128	5.30
**9**	alprazolam	97	4.02
**11**	sertraline	88	3.64
**17**	fluoxetine	65	2.69
**20**	bromazepam	48	1.99
**23**	citalopram	42	1.74
**27**	duloxetine	37	1.53
**29**	prazepam	37	1.53
**32**	aripiprazole	35	1.45
**33**	diazepam	35	1.45
**42**	mirtazapine	26	1.08
**43**	clomipramine	25	1.04
**46**	mianserine	25	1.04
**47**	amitriptyline	24	0.99
**48**	oxazepam	24	0.99
**51**	amisulpride	22	0.91
**56**	agomelatin	21	0.87
**63**	hydroxyzin	20	0.83
**64**	risperidon	20	0.83
**66**	lorazepam	19	0.79
**74**	cyamemazin	17	0.70
**79**	olanzapine	16	0.66
**81**	quetiapin	16	0.66
**86**	etifoxin	14	0.58
